# Primary Patency Success of Arteriovenous Shunts in Hemodialysis Patients: A 28-Month Prospective Study

**DOI:** 10.7759/cureus.70206

**Published:** 2024-09-25

**Authors:** Nabil Al_Madhwahi, Zaid Al-Dailami, Gehad AL-Mashramah, Haitham M Jowah

**Affiliations:** 1 Department of Vascular Surgery, Faculty of Medicine and Health Science, Sana’a University, Sana’a, YEM; 2 Department of Vascular Surgery, Al-Thawra Modern General Hospital, Sana'a, YEM; 3 Department of Orthopedic Surgery, Al-Thawra Modern General Hospital, Sana'a, YEM; 4 Department of Surgery, Faculty of Medicine and Health Science, Sana'a University, Sana'a, YEM

**Keywords:** arteriovenous shunts, brachiocephalic shunts, hemodialysis, primary patency, radiocephalic shunts, yemen

## Abstract

Background

Arteriovenous (AV) shunts are vital for providing long-term vascular access in hemodialysis patients. While brachiocephalic and radiocephalic shunts are commonly employed, data on their primary patency rates and associated complications in resource-limited settings such as Yemen remain scarce. This study aimed to evaluate the primary patency success of AV shunts and identify factors influencing their outcomes in hemodialysis patients at Al-Thawra Modern General Hospital.

Methods

This prospective observational study was conducted over 28 months, from April 2021 to August 2023, at Al-Thawra Modern General Hospital in Sana’a, Yemen. A total of 163 patients with chronic renal failure requiring AV shunt creation for hemodialysis were included. Data on patient demographics, comorbidities, shunt characteristics, and postoperative outcomes were collected. Primary patency was assessed at the 8-month follow-up interval. Statistical analysis was performed to identify factors associated with shunt patency.

Results

Among the enrolled 163 patients, the median age was 43.12 years, with 61.3% undergoing brachiocephalic shunt creation. The overall primary patency rate at 8 months was 87.7%, with brachiocephalic shunts exhibiting a significantly greater patency rate (93.0%) than radiocephalic shunts (67.3%) (p = 0.02). Complications occurred in 25% of patients, with pseudoaneurysm formation being the most common complication (6.1%). Factors such as shunt type, patient age, and comorbidities (e.g., diabetes) significantly influence patency outcomes.

Conclusion

Compared with radiocephalic shunts, brachiocephalic shunts demonstrated superior primary patency rates, suggesting that they may be a preferable option for long-term hemodialysis access in the studied population. These findings emphasize the need for individualized patient management and careful postoperative monitoring, particularly in resource-limited settings such as Yemen, to optimize AV shunt outcomes.

## Introduction

Compared with other vascular access options, arteriovenous fistulas (AVFs) are preferred for long-term hemodialysis access because of their superior patency rates and lower risk of complications, such as grafts and central venous catheters [1]. In regions such as Yemen, where healthcare resources are limited, the importance of effective and durable vascular access is paramount as the burden of end-stage renal disease (ESRD) continues to increase. Yemen faces a growing incidence of ESRD, with cases rising from an estimated 93 per million people in 2006 to over 5200 patients on maintenance dialysis by 2019 [2]. The rapid increase places significant strain on the already overburdened healthcare system, highlighting the urgent need for reliable and sustainable access to dialysis.

Despite the critical role of AVFs in ensuring successful hemodialysis, data from low- and middle-income countries, including Yemen, on AVF outcomes are lacking [3]. The absence of such data hinders the development of evidence-based strategies to improve patient outcomes, particularly in healthcare systems that are already stretched to their limits. Existing studies have primarily focused on outcomes in high-resource settings, where factors such as patient selection, surgical expertise, and postoperative care differ significantly from those in Yemen. This gap in the literature underscores the need for region-specific research to inform local clinical practices and policies.

In regards to the Yemeni population, brachiocephalic shunts are the most commonly used AVFs, followed by radiocephalic and brachiobasilic shunts [4]. These findings are consistent with international practices, although specific preferences may vary depending on local expertise and patient characteristics [5]. However, the primary patency rates and factors influencing these outcomes in Yemeni patients remain poorly understood. Accurate preoperative assessment and careful selection of the AVF site are essential to optimize the success of these procedures and minimize complications [6].

This study addresses the pressing need for data on AVF outcomes in Yemeni patients undergoing hemodialysis. By evaluating the primary success rates and identifying factors that influence AVF patency at the Al-Thawra Modern General Hospital in Sana’a, this study provides critical insights that may benefit clinical practice and healthcare policy. The findings of this study are expected to not only fill a significant gap in the literature but also guide efforts to enhance dialysis care in resource-limited settings, such as Yemen, ultimately contributing to improved patient outcomes and reduced healthcare burdens.

## Materials and methods

Study design

This prospective observational study was conducted at the Al-Thawra Modern General Hospital in Sana’a, Yemen, over a 28-month period from April 2021 to August 2023. The current study primarily aimed to evaluate the primary patency success rates of arteriovenous (AV) shunts in patients with chronic renal failure undergoing hemodialysis. Additionally, this study aimed to identify patient- and shunt-related factors that influence patency outcomes.

Study population

The study population comprised 163 patients diagnosed with chronic renal failure who required AV shunt creation for hemodialysis. The inclusion criteria were patients on maintenance hemodialysis who required AV shunt placement and who provided informed consent. Patients who refused participation and were lost to follow-up or had peripheral vascular or extensive arterial occlusive disease were excluded to ensure an accurate assessment of AV shunt outcomes.

Preoperative assessment

All patients underwent a comprehensive preoperative evaluation, including a physical examination to assess arterial pulses and venous outflow in the upper extremities. Doppler ultrasonography was routinely performed to evaluate the suitability of the radial or brachial arteries for vascular access creation, with measurements including artery diameter, systolic flow rate, and resistive index. These assessments are crucial to predict postoperative success and optimize the selection of the AVF site.

Surgical procedure

All AV shunts were created under moderate sedation with local anesthesia (0.5% lidocaine). For brachiocephalic shunts, a transverse incision was made at or just below the elbow crease, and the cephalic vein was mobilized and flushed with heparinized saline. An end-to-side anastomosis to the brachial artery was performed using 6-0 polypropylene sutures. For radiocephalic shunts, a longitudinal incision was made at the wrist, and the cephalic vein was anastomosed to the radial artery in a similar fashion. In cases where intraoperative complications, such as poor vessel quality or bleeding, occurred, decisions were made intraoperatively by the senior surgeon to either change the anastomosis site or utilize alternative vascular clamps and sutures. Intraoperative patency was confirmed via a non-directional Doppler probe.

Postoperative assessment and follow-up

Postoperative evaluations were conducted on the first and seventh days after surgery, with follow-up continuing for a maximum of eight months. These evaluations included a physical examination to check for a thrill and pulse at the fistula site, as well as Doppler ultrasonography to measure fistula size, velocity, volume flow, and resistive index of the distal artery. The postoperative and preoperative values were compared to determine shunt functionality and detect any early complications.

Data collection

Data were collected via a structured questionnaire that included demographic information, clinical data, and outcome measures. Demographic data included age, sex, and medical history, whereas clinical data focused on the duration of hemodialysis, previous AV shunt placements, comorbidities, and surgical procedures. The outcome measures included primary patency rates, postoperative complications, and reintervention rates to provide a comprehensive assessment of AV shunt success and durability.

Statistical analysis

The data were processed using IBM Corp. Released 2017. IBM SPSS Statistics for Windows, Version 25.0. Armonk, NY: IBM Corp. Descriptive statistics were employed to summarize patient demographics and clinical characteristics, with categorical variables presented as frequencies and percentages and continuous variables summarized using means and standard deviations. Associations between categorical variables were assessed using the chi-square test, whereas multiple response analysis was applied to capture the complexity of patient outcomes in this diverse population. Statistical significance was set at p < 0.05.

Ethical considerations

The study was conducted in accordance with the ethical principles outlined in the Declaration of Helsinki. Ethical approval for this study was obtained from the Ethical Committee of Al-Thawra Modern General Hospital, Institutional Review Board (IRB) approval number TMGH/IRB/2021/022, which was granted on March 15, 2021. All participants provided informed consent after being fully informed of the study objectives, procedures, potential risks, and benefits. The participants were assured of the voluntary nature of their involvement and the confidentiality of their personal information.

## Results

Demographic and baseline characteristics

The study included 163 patients with chronic renal failure who underwent AV shunt creation for hemodialysis. The median age was 43.12 years, with a range from 9 to 80 years. The majority of patients (33.7%) were aged between 41 and 60 years, and 55.8% of the patients were female. Common comorbidities included diabetes mellitus (50.3%) and hypertension (34.4%). A significant portion of the patients (68.1%) received their first AV shunt, whereas 31.9% had a history of previous vascular access, indicating a more complex clinical background for this subset of patients (Table 1).

**Table 1:** Demographics and baseline characteristics of the patients (n = 163)

Characteristic	Frequency (n = 163)	Percentage (%)
Age (years)		
Median, IQR	43.12 (9-80)	-
Mean ± SD	43.12 ± 15.46	-
Age group (years)		
9–20	22	13.5
21–40	49	30.1
41–60	55	33.7
61–80	37	22.7
Gender		
Male	72	44.2
Female	91	55.8
Comorbidities		
Diabetes Mellitus	82	50.3
Hypertension	56	34.4
Coronary Artery Disease	24	14.7
Peripheral Vascular Disease	18	11.0
Chronic Obstructive Pulmonary Disease	12	7.4
History of previous vascular access		
Yes	111	68
No	52	32

AV shunt characteristics

The most common site for AV shunt creation was the left cubital fossa (49.7%), followed by the left wrist (29.1%). Brachiocephalic shunts were the most frequently performed shunts (61.3%), followed by radiocephalic shunts (32%), and brachiobasilic shunts (6.7%). These findings are consistent with the established preference for brachiocephalic shunts in this population because of their relatively high patency rates and suitability for long-term dialysis access (Table 2).

**Table 2:** AV shunt characteristics (n = 163)

Characteristics	Frequency	Percentage (%)
Shunt Location		
Left Cubital Fossa	81	49.7
Left Wrist	47	29.1
Right Cubital Fossa	25	15.1
Right Wrist	10	6.1
Shunt Type		
Brachiocephalic	100	61.3
Radiocephalic	52	32
Brachiobasilic	11	6.7

Complications and outcomes

Approximately 25% of the patients experienced complications during the study period. The most common complication was pseudoaneurysm formation (6.1%), followed by infection at the fistula site (4.9%), venous hypertension (4.9%), thrombosis (3.7%), and aneurysm rupture (3.1%). These complications were managed conservatively or surgically, depending on their severity and impact on shunt functionality (Table 3).

**Table 3:** Complications associated with AV shunts among study participants (n = 163)

Complication	Frequency	Percentage (%)
Pseudoaneurysm	10	6.1
Infection at the Fistula Site	8	4.9
Venous Hypertension	8	4.9
Thrombosis	6	3.7
Aneurysm Rupture	5	3.1

Primary patency outcomes

At the eight-month follow-up, 87.7% of the shunts (143 out of 163) remained patent, whereas 12.3% (20 out of 163) were not patent. Among the 20 nonpatent shunts, 80% underwent revision, and 20% required the creation of a new shunt. When the distribution of patent shunts by type was examined, the majority were brachiocephalic (65%), followed by radiocephalic (29%) and brachiobasilic (6.3%). These results highlight the superior performance of brachiocephalic shunts in maintaining long-term functionality compared with other anatomical access (Table 4).

**Table 4:** Primary shunt patency and reintervention outcomes (n = 163)

Variable	Frequency	Percentage (%)
Primary Shunt Patency		
Patent	143	87.7%
Not Patent	20	12.3%
Reintervention Among Non-Patent Shunts (n = 20)		
Shunt Revision	16	80.0%
New Shunt Creation	4	20.0%
Distribution of Patent Shunts by Shunt Type (n = 143)		
Brachiocephalic	93	65.0%
Radiocephalic	41	29.0%
Brachiobasilic	9	6.3%

Overall, the primary patency rate across all shunt types was 87.7%. Brachiocephalic shunts had the highest patency rate (93.0%), followed by brachiobasilic shunts (81.8%) and radiocephalic shunts (67.3%). These findings underscore the superior outcomes associated with brachiocephalic shunts, making them a preferable choice for long-term hemodialysis access in this patient population (Table 5).

**Table 5:** Patency rates by shunt type

Shunt Type	Patent Shunt (n = 143)	Total Shunt (n = 163)	Patency Rate (%)
Overall Primary Patency	143	163	87.7%
Brachiocephalic	93	100	93.0%
Brachiobasilic	9	11	81.8%
Radiocephalic	35	52	67.3%

Factors influencing primary patience

At the eight-month follow-up, primary patency rates were analyzed for various factors, including age, comorbidities, and shunt type. The 41-60-year-old age group had the highest proportion of patent shunts (34.3%), whereas the 21-40 and 61-80-year-old age groups also presented relatively high patent rates (30.1% and 22.4%, respectively). Comorbidities, such as diabetes and hypertension, were common among patients with patent shunts (49.7% and 33.6% of this group, respectively). However, the most significant factor influencing the patency rate was the type of shunt used. Brachiocephalic shunts had the highest patency rate, with 65.0% remaining patent, whereas radiocephalic shunts had a higher failure rate, with 55.0% failing (p = 0.02). This result indicates that shunt type is a critical determinant of long-term patency outcomes (Table 6).

**Table 6:** Factors influencing primary patience rates during the eight-month follow-up Note: Chi-square values for each factor are as follows: Age Group = 0.19, Comorbidities = 0.28, and Shunt Type = 6.75. * Statistical significance was assessed using the chi-square test. A p-value of <0.05 was considered significant.

Factor	Patent, n =143 (87.7%)	Failed, n = 20 (12.3%)	p-value
Age Group (years)			0.15
9–20	19 (13.3%)	3 (15.0%)	
21–40	43 (30.1%)	6 (30.0%)	
41–60	49 (34.3%)	6 (30.0%)	
61–80	32 (22.4%)	5 (25.0%)	
Comorbidities			0.22
Diabetes Mellitus	71 (49.7%)	11 (55.0%)	
Hypertension	48 (33.6%)	8 (40.0%)	
Coronary Artery Disease	20 (14.0%)	4 (20.0%)	
Peripheral Vascular Disease	15 (10.5%)	3 (15.0%)	
COPD	10 (7.0%)	2 (10.0%)	
Shunt Type			0.02*
Brachiocephalic	93 (65.0%)	7 (35.0%)	
Brachiobasilic	9 (6.3%)	2 (10.0%)	
Radiocephalic	41 (28.7%)	11 (55.0%)	

## Discussion

This prospective study evaluated the primary patency success of arteriovenous (AV) shunts in patients undergoing hemodialysis at Al-Thawra Modern General Hospital. Our findings indicated a high primary patency rate of 87.7% at eight months of follow-up, with brachiocephalic shunts exhibiting the highest patency rate (93.0%), significantly outperforming radiocephalic shunts (67.3%) (p = 0.02). These results are consistent with several studies that emphasize the superiority of brachiocephalic AV fistulas for long-term hemodialysis access. For instance, Nguyen et al. (2007) reported faster maturation times and superior long-term patency for brachiocephalic fistulas (16 months) compared to radiocephalic fistulas (13 months), highlighting the advantages of brachiocephalic AVFs in providing reliable access for hemodialysis [7]. Similarly, Ahmad et al. (2022) observed significantly higher patency rates in brachiocephalic AVFs (97%) compared to radiocephalic AVFs (80.5%) [8]. These findings further support the recommendation to prioritize brachiocephalic shunts for patients requiring long-term vascular access, particularly in resource-limited settings like Yemen.

Wong et al. (2021) found comparable overall patency rates between brachiocephalic and radiocephalic shunts but noted that patient selection and surgical expertise play crucial roles in achieving successful outcomes [9]. Additionally, a recent comparison study between patients with and without diabetes confirmed that brachiocephalic shunts offer superior primary patency compared to radiocephalic shunts, particularly in diabetic populations, which mirrors our findings of a patency rate of 93.0% for brachiocephalic shunts. This supports the notion that brachiocephalic shunts are more resilient, especially in populations with comorbidities such as diabetes [10].

Although our study focused on primary patency rates at the eight-month follow-up, it is important to consider long-term patency outcomes beyond this period. Knežević et al. (2022) suggested that the use of antiplatelet medications during the early postoperative period (30-90 days) may enhance long-term patency, while Gangadharan et al. (2021) highlighted the role of technical factors such as maturation time and dialysis sessions in maintaining patency over longer periods [11,12]. Furthermore, interventions such as percutaneous transluminal angioplasty (PTA) have been shown to improve secondary cumulative patency rates for radiocephalic fistulas, although the statistical significance remains debated [13]. Future studies should explore the long-term durability of these interventions in resource-limited settings to further optimize patient outcomes.

Moreover, a study comparing the long-term patency of brachiocephalic and radiocephalic shunts over a 24-month period reported that brachiocephalic fistulas maintained a higher patency rate (58.8% vs. 49.1%), further reinforcing the advantages of this shunt type for extended use [14]. However, while brachiocephalic shunts may offer better patency, they are also associated with higher risks of complications such as steal syndrome and edema [15,16]. These findings underscore the need for careful patient selection and postoperative monitoring to balance the benefits of higher patency rates against the increased risks.

In our study, 25% of patients experienced complications, with pseudoaneurysm formation being the most common (6.1%), followed by infection (4.9%), venous hypertension (4.9%), and thrombosis (3.7%). These findings align with those reported by Bode (2012), which emphasizes that brachiocephalic AV fistulas, despite their better patency, carry a higher risk of complications [17]. Additionally, Stolić et al. (2012) and Zandieh et al. (2014) reported that stenosis and neointimal hyperplasia are frequent causes of AV access failure [18,19]. Other studies have emphasized the importance of early detection and management of such complications to prevent further deterioration of access function [20]. This is particularly relevant in resource-limited environments, where access to specialized vascular care may be constrained. Close monitoring and timely interventions are essential to preserve shunt functionality, as also noted by Connolly et al. (1984) [21].

Our study identified several factors that influence primary patency outcomes, consistent with prior research. Patient-related factors such as advanced age and comorbidities, particularly diabetes, were shown to have a significant impact on patency. Fitzgerald et al. (2004) and Smith et al. (2012) reported that diabetes, hypertension, and peripheral vascular disease are associated with longer maturation times and lower patency rates [22,23]. In our cohort, 49.7% of patients with patent shunts had diabetes, suggesting that brachiocephalic shunts may be more resilient in such populations. Additionally, our finding that patients aged 41-60 had the highest proportion of patent shunts (34.3%) aligns with research indicating that middle-aged patients may benefit the most from AV shunt placement, while older patients tend to have poorer patency due to comorbidities [23]. Other patient characteristics such as tobacco use, small vessel caliber, and previous access failure have also been shown to negatively affect shunt outcomes [12].

Effective postoperative care not only determines the success of AV shunts for hemodialysis but also plays a crucial role. Studies by Whittier (2009) and Flu et al. (2008) emphasize the importance of implementing surveillance protocols, including clinical monitoring, flow measurements, and duplex ultrasound imaging, to detect complications like stenosis early [24,25]. In resource-limited settings, optimized care protocols involving a multidisciplinary team can significantly enhance both primary and secondary patency rates, reducing the need for surgical revisions [25]. Color duplex imaging and minimally invasive venography are also crucial for visualizing stenosis, which is the leading cause of access failure [24]. A collaborative approach involving nephrologists, vascular surgeons, and dialysis nurses, as well as regular clinical surveillance, is essential to maintaining shunt functionality and improving long-term outcomes [26,27].

In conclusion, our study demonstrated that brachiocephalic shunts provide superior primary patency rates compared with radiocephalic shunts in patients undergoing hemodialysis, making them the preferred option for long-term vascular access, especially in resource-limited settings such as Yemen. The high primary patency rate (87.7%) observed in this study underscores the effectiveness of AV shunts in maintaining long-term functionality, particularly when brachiocephalic shunts are used. However, the significant complication rates, particularly the formation of pseudoaneurysms and other vascular complications, highlight the necessity for diligent postoperative monitoring and timely intervention. These findings emphasize the importance of individualized patient management, considering factors such as age, comorbidities, and shunt type, to optimize outcomes and minimize complications.

Given the challenges facing healthcare systems in low- and middle-income countries, this study provides valuable insights that could inform local clinical practices and policies. Prioritizing brachiocephalic shunts and implementing rigorous follow-up protocols may increase the success rate of hemodialysis programs in similar contexts. Future studies with longer follow-up periods and multicenter involvement are recommended to validate these findings and further explore the long-term durability and outcomes of AV shunts in diverse patient populations. By addressing these gaps, future research can contribute toward improving the quality of dialysis care and ultimately reducing the burden of ESRD in resource-limited settings.

Strengths and limitations of our study

A strength of our study is its prospective design, which allowed for systematic data collection and follow-up, thereby ensuring the reliability of the observed outcomes. Additionally, the relatively large sample size for a single-center study enhances the generalizability of our findings in similar settings with limited resources. However, our study is not without limitations. The single-center design may limit the generalizability of our results to other settings with different patient populations or healthcare infrastructures. Furthermore, a follow-up period of 8 months, while sufficient for assessing early patency, may not capture long-term outcomes, which are crucial for understanding the durability of AV shunts. Further studies with longer follow-up periods and multicenter designs are recommended to validate our findings and explore the factors influencing long-term patency.

## Conclusions

This study demonstrated that brachiocephalic shunts have superior primary patency rates compared to radiocephalic shunts, making them the preferred choice for long-term vascular access in patients undergoing hemodialysis, particularly in resource-limited settings like Yemen. With an overall primary patency rate of 87.7%, brachiocephalic shunts offer significant advantages, though complications such as pseudoaneurysms and venous hypertension necessitate diligent postoperative monitoring. The findings underscore the importance of individualized patient management and prompt interventions to maintain shunt functionality. Further studies with longer follow-up periods and multicenter involvement are recommended to validate these outcomes and improve dialysis care in similar contexts.
